# Bandstructure and Size-Scaling Effects in the Performance of Monolayer Black Phosphorus Nanodevices

**DOI:** 10.3390/ma15010243

**Published:** 2021-12-29

**Authors:** Mirko Poljak, Mislav Matić

**Affiliations:** Computational Nanoelectronics Group, Faculty of Electrical Engineering and Computing, University of Zagreb, HR 10000 Zagreb, Croatia; mislav.matic@fer.hr

**Keywords:** black phosphorus, phosphorene, nanoribbon, bandstructure, quantum transport, NEGF, nanodevice, field-effect transistor, scaling, average charge velocity

## Abstract

Nanodevices based on monolayer black phosphorus or phosphorene are promising for future electron devices in high density integrated circuits. We investigate bandstructure and size-scaling effects in the electronic and transport properties of phosphorene nanoribbons (PNRs) and the performance of ultra-scaled PNR field-effect transistors (FETs) using advanced theoretical and computational approaches. Material and device properties are obtained by non-equilibrium Green’s function (NEGF) formalism combined with a novel tight-binding (TB) model fitted on ab initio density-functional theory (DFT) calculations. We report significant changes in the dispersion, number, and configuration of electronic subbands, density of states, and transmission of PNRs with nanoribbon width (*W*) downscaling. In addition, the performance of PNR FETs with 15 nm-long channels are self-consistently assessed by exploring the behavior of charge density, quantum capacitance, and average charge velocity in the channel. The dominant consequence of *W* downscaling is the decrease of charge velocity, which in turn deteriorates the ON-state current in PNR FETs with narrower nanoribbon channels. Nevertheless, we find optimum nanodevices with *W* > 1.4 nm that meet the requirements set by the semiconductor industry for the “3 nm” technology generation, which illustrates the importance of properly accounting bandstructure effects that occur in sub-5 nm-wide PNRs.

## 1. Introduction

**Figure 1:**
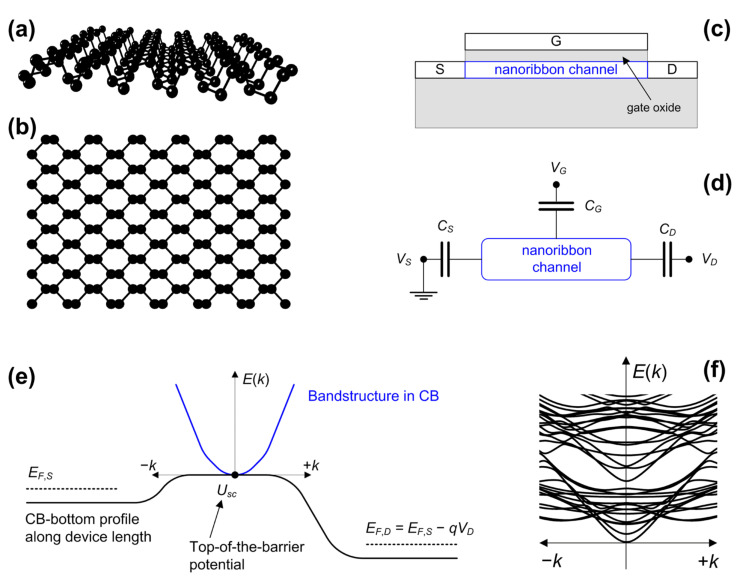
Illustration of a phosphorene nanoribbon with (**a**) side and (**b**) top view, as well as of (**c**) PNR FET cross-sectional view. ToB model description with (**d**) capacitive model, (**e**) bandstructure along the channel length, and (**f**) example of a PNR bandstructure calculated by the DFT-TB model.

Monolayer black phosphorus (BP) or phosphorene, illustrated in Figure 1a,b, is a promising two-dimensional (2D) material for the realization of future electron devices in integrated circuits due to its favorable electronic and transport properties, which include acceptable bandgap and carrier mobility [1,2]. Large-area BP field-effect transistors (FETs) have been experimentally demonstrated [2,3,4], and short-channel and wide-gate phosphorene FETs have been theoretically studied [5,6]. On the other hand, patterning monolayer BP into phosphorene nanoribbons (PNRs) provides a technologically relevant way of adjusting the electronic and transport properties by quantum confinement effects [7,8,9,10]. Ultra-narrow PNRs have been fabricated and characterized recently with widths down to ~0.5 nm [11,12], which further kindles interest in theoretical research of PNR-based nanoelectronic devices.

For a proper assessment of these nanostructured phosphorene FETs, methodology must be based on advanced theoretical formalisms such as quantum transport, e.g., non-equilibrium Green’s function (NEGF) formalism, because the transport physics in nanodevices is governed by quantum effects [13,14,15]. Moreover, these nanostructures must be described by proper atomically-resolved Hamiltonians that consider the complex bandstructure of such materials at the nanoscale [16,17]. The best bandstructure description is naturally provided by ab initio methods such as density-functional theory (DFT) calculations. However, the Hamiltonians obtained by DFT are very large and dense matrices which makes DFT Hamiltonians difficult to implement for quantum transport simulations for realistically sized FETs [5,17,18]. Tight-binding (TB) models are more readily applicable and result in more computationally efficient NEGF simulations. A TB model from the relevant literature (TBL model) for phosphorene, as described in [19], is widely used to explore the properties of BP and PNRs [9,20,21,22]. Nevertheless, in comparison to DFT results [23], the TBL model does not reproduce the intricate multi-valley bandstructure of PNRs with the widths under ~5 nm.

In [24,25], we introduced a new DFT- based TB Hamiltonian model (DFT-TB model) that describes the bandstructure of ultra-narrow PNRs more accurately. The DFT-TB model is more complicated than the TBL model, with larger and denser unit-cell and coupling matrices but is still numerically efficient in comparison to coupled DFT-NEGF simulations. In this paper, we employ NEGF and the DFT-TB model to study size-scaling and bandstructure effects in the electronic and transport properties of PNRs, and the performance of ballistic PNR FETs with nanoribbon widths under 5 nm. We show that PNR width downscaling significantly modifies the dispersion, including the bandgap and effective mass, together with a considerable impact on density of states and transmission through the nanoribbon. For PNR FETs, we demonstrate the deterioration of the ON-state current with decreasing PNR width; however, a surprising maximum of the width-normalized current is reported for ~2.5 nm-wide PNR FET. These findings are further investigated by examining the width- and bias-dependence of channel charge density and average charge velocity. We find that width-scaling-induced bandstructure effects in carrier velocity behavior are the dominant factor that determines the properties of PNR FET current driving capabilities in the ON-state.

## 2. Methods

### 2.1. DFT-Based Tight-Binding Hamiltonian

Due to the limitations of the TBL Hamiltonian model, especially in describing ultra-narrow PNRs, this work uses the recently developed DFT-TB model, which reproduces all important size-scaling effects on the bandstructure of PNRs [24,25]. In the development of the new DFT-TB model, the DFT simulations were performed using the OpenMX package [26,27], employing generalized gradient approximation (GGA) with Perdew–Burke–Ernzerhof (PBE) exchange-correlation (XC) functional. The DFT results were used as inputs into TBStudio [28], a new software package that implements the Slater–Koster (SK) method, and allows the choice of orbital number and type for fitting the DFT data [29]. In the new DFT-TB model, 4 orbitals are included for each phosphorus atom (*s*, *p_x_*, *p_y_* and *p_z_*), and all relevant SK overlap integrals are accounted for (*ssσ*, *spσ*, *ppσ*, *ppπ*). We showed in [24,25] that our new DFT-TB model achieves excellent agreement with DFT bandstructure in the energy range of interest, i.e., within ~1 eV from the conduction band minimum (CBM) and valence band maximum (VBM). The model data needed for the construction of PNR Hamiltonians can be found in Supplementary Materials. Electronic properties, transport properties, and PNR FET performance obtained using the new DFT-TB model are compared to those obtained with a simpler widely-used TBL model [19], in order to demonstrate the strong impact of bandstructure effects. These two TB Hamiltonians are used to study width-dependent dispersion of ultra-scaled PNRs and as inputs in the NEGF equations, which enables the investigation of the transport properties of PNRs and the performance of PNR FETs.

### 2.2. Quantum Transport with NEGF

The NEGF formalism is utilized in this work to solve the Schrödinger’s equation with open boundary conditions (OBCs). As will be explained later in Section 2.3, we need the transmission function and density of states of the PNR, so that only the equilibrium part of our in-house NEGF code for 2D material nanostructures [30,31] is needed. The central quantity of the NEGF formalism is the retarded Green’s function of the device and is obtained by

(1) $$G^{R}(E)={\left[\left(E+i0^{+}\right)I-H-\Sigma_{S}^{R}(E)-\Sigma_{D}^{R}(E)\right]}^{-1}$$

where *H* is the device Hamiltonian constructed using either the new DFT-TB or the existing TBL model. The size and sparsity of the Hamiltonian matrix depends on the model used, as well as on nanoribbon width (*W*) and length (*L*). The Σ*^R^* matrices are the retarded contact self-energies that consider the OBCs to the two contacts (source, *S*, and drain, *D*). These self-energy matrices are found by the iterative and numerically efficient Sancho–Rubio method [32]. The NEGF calculations in this work assume ideal contacts, meaning that the *S*/*D* extensions or reservoirs are semi-infinite semiconducting PNRs. This choice is common in the literature as it eliminates noncoherent effects at the channel-contact interfaces, and introduces no additional contact resistance into the nanostructure [33]. The retarded (*G^R^*) and advanced Green’s function (*G^A^*) of the device, where *G^A^* = *G^R^*^†^, are then used to find the transmission function, *T*(*E*), and density of states, *DOS*(*E*), according to expressions given in e.g., [9,34,35].

### 2.3. Top-of-the-Barrier Device Model

The characteristics of ultra-scaled PNR FETs, illustrated in Figure 1c, and relevant device performance metrics are obtained using the top-of-the-barrier (ToB) device model, which self-consistently solves the Poisson equation that exploits the NEGF results and provides ballistic device characteristics [22,36,37]. The ToB model does not include tunneling, so its relevance is limited to the above-threshold operation region and can be utilized to adequately assess the ON-state performance in devices with channel lengths larger than ~10 nm [36,37].

In the ToB model, the central parameter is the top-of-the-barrier potential (*U_sc_*), located at the maximum of the source-drain barrier. Assuming a grounded source, the capacitive model defined in Figure 1d results in the following potential:

(2) $$U_{sc}=U_{sc0}-q\left(\alpha_{G}V_{GS}+\alpha_{D}V_{DS}\right)+\frac{q}{C_{ox}}\left(Q_{s}-Q_{s}(V_{GS}=0)\right)$$

where the *α* parameters describe the capacitive coupling between the electrodes and the channel, e.g., gate coupling is defined as *α_G_* = *C_G_*/(*C_S_* + *C_D_* + *C_G_*). We set *α_G_* = 1 and *α_D_* = 0, assuming that the gate electrode exhibits ideal control over the atomically-thin nanoribbon channel. In (2), *C_ox_* is the gate oxide capacitance, and *Q_s_* is the inversion charge density in the channel at top-of-the-barrier. The *Q_s_* depends on the positions of *U_sc_* and Fermi levels in source and drain regions (*E_F_*_,*S*_ and *E_F_*_,*D*_, respectively), which are shown in Figure 1e, and is found as follows:

(3) $$Q_{s}=Q_{s,S}+Q_{s,D}$$

(4) $$Q_{s,S(D)}=\frac{1}{2}\int_{-\infty }^{+\infty }DOS(E-U_{sc})f(E-E_{F,S(D)})dE$$

with *E_F_*_,*D*_ = *E_F_*_,*S*_ − *qV_DS_*. After convergence is achieved for *U_sc_* and *Q_s_*, the drain current is calculated using the Landauer formula [34]. In all calculations, we assume that devices operate at room temperature, i.e., *T* = 300 K; studying temperature-related effects, including self-heating, is beyond the scope of this work.

The charge density defined in (4) and the Landauer’s current formula take as inputs the *DOS*(*E*) and *T*(*E*), respectively, which are found by the NEGF simulations. Therefore, despite its relative simplicity, the ToB device model inherently includes all size-scaling and bandstructure-related effects with atomistic and orbital resolution. A simplified single-band effective-mass bandstructure in the conduction band is shown in Figure 1e, whereas our results are based on TB Hamiltonians, with an example of PNR dispersion obtained with the DFT-TB model shown in Figure 1f. Therefore, the described approach will allow us to explore accurately the impact of bandstructure and size scaling on the performance of ballistic PNR FETs.

## 3. Results and Discussions

### 3.1. Electronic and Transport Properties of Ultra-Narrow PNRs

**Figure 2:**
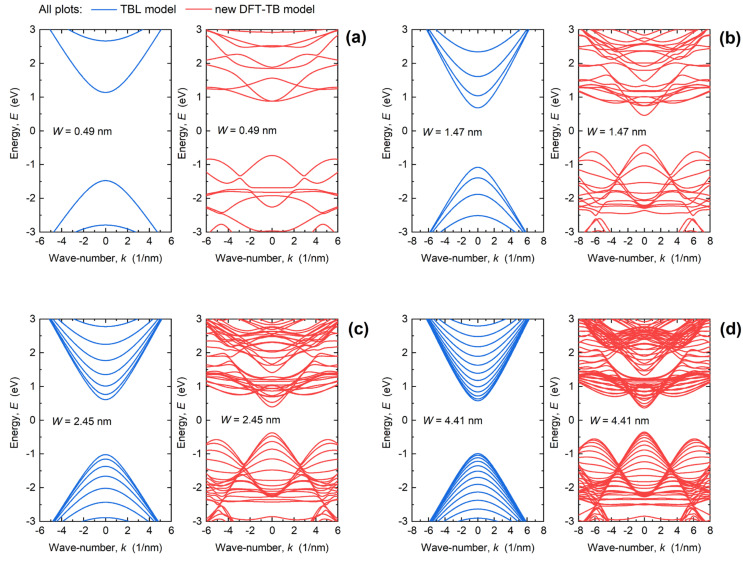
Comparison of dispersions obtained by the DFT-TB (panels on the right) and TBL (panels on the left) models for PNR widths of (**a**) 0.49 nm, (**b**) 1.47 nm, (**c**) 2.45 nm, and (**d**) 4.41 nm.

Figure 2 reports the dispersion curves for various PNRs, with W ranging from 0.49 nm to 4.41 nm, calculated using the TBL and DFT-TB Hamiltonians. We observe that the DFT-TB Hamiltonian results in multi-valley bandstructure in both the conduction and valence band, which agrees with DFT studies of ultra-narrow PNRs reported in [23,38]. In contrast, the TBL Hamiltonian produces a symmetric single valley for both electrons and holes in the conduction and valence bands, respectively, irrespective of PNR width. Therefore, we expect a more accurate analysis of electronic, transport and device properties of PNRs and PNR FETs by using the DFT-TB model.

With downscaling the PNR width from 4.41 nm to 0.49 nm, bandgap (*E_G_*) increases considerably for both TB models, i.e., from 1.57 eV to 2.61 eV (TBL) and from 0.71 eV to 1.61 eV (DFT-TB). The TBL model provides wider bandgaps due to different XC functionals used for the development of the two Hamiltonian models. Namely, PBE was used for our DFT-TB model [24,25], which results in a lower *E_G_* in comparison to the HSE functional used for the development of the TBL model [19]. The PBE functionals are known to underestimate the bandgap, so the realistic *E_G_* value is expected to be between those obtained by PBE and HSE DFT simulations. Nevertheless, our DFT-TB model provides more accurate dispersion properties for both electrons and holes. Since the ToB device model relies on the bandstructure properties not far away from CBM and VBM, we avoid artificial *E_G_* adjustment in the DFT-TB model.

Due to the larger number of orbitals considered in the DFT-TB model, the related bandstructure plots in Figure 2 contain a larger number of subbands and, hence, conducting modes than those obtained by the TBL model. Consequently, NEGF simulations using the DFT-TB model should result in higher transmission probabilities at the same energy away from the CBM or VBM in comparison to the TBL results. When PNR width is scaled down, the number of subbands decreases for both models. However, improved bandstructure description by the DFT-TB model shows that even the narrowest PNRs, with *W* of 0.49 nm (Figure 2a) and 1.47 nm (Figure 2b), exhibit a much richer dispersion so that higher transmission probabilities and more enhanced DOS are expected in these devices than predicted by the TBL model.

Regarding carrier effective masses (m*), in wider PNRs, both TB models provide similar m* values near the CBM and VBM, while the difference increases considerably when the width is scaled down. For *W* = 4.41 nm, m* in the first subband equals ~0.28*m*_0_ (TBL) and ~0.21*m*_0_ (DFT-TB). For *W* = 0.49 nm, the DFT-TB model results in much heavier electrons in comparison to the TBL model. Namely, the DFT-TB model gives m*~1.9*m*_0_ in the first subband and m*~0.5*m*_0_ in the second subband, while the TBL model results in m*~0.5*m*_0_ in the first subband. The lower dispersion curvature, i.e., heavier carriers, in either the conduction or valence band generally leads to increased DOS that benefits inversion charge density in the channel. At the same time, heavier carriers exhibit diminished carrier velocities, which negatively impacts the current drivability of PNR FETs. Due to interplay of different phenomena, from subband number to effective mass change, it is difficult to qualitatively estimate how the PNR width downscaling will impact device performance purely from analyzing the dispersions.

**Figure 3:**
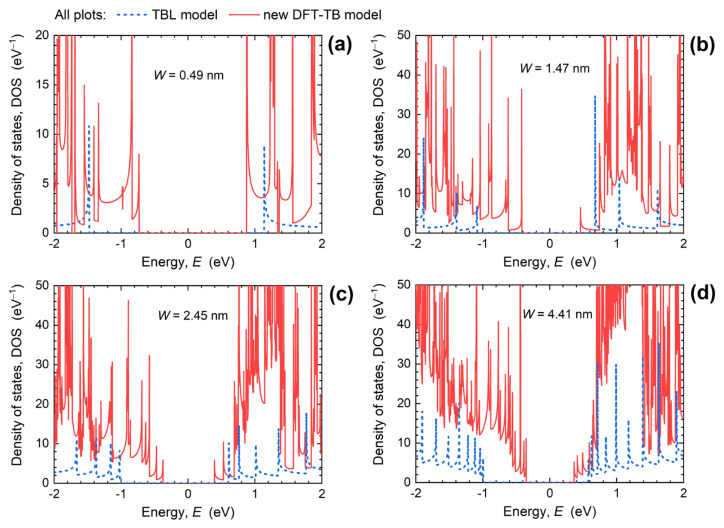
Density of states in PNRs obtained using the DFT-TB and TBL models for PNR widths of (**a**) 0.49 nm, (**b**) 1.47 nm, (**c**) 2.45 nm, and (**d**) 4.41 nm.

Size-scaling effects observed in dispersion characteristics in Figure 2 consequently have a strong impact on the DOS and transmission of ultra-narrow PNRs. Figure 3 shows the DOS obtained with DFT-TB and TBL models for PNR widths of 0.49 nm, 1.47 nm, 2.45 nm, and 4.41 nm. When *W* is scaled down, DFT-TB DOS generally decreases away from the CBM and VBM due to reduced number of subbands in narrower nanoribbons. The two TB models provide approximately equal DOS in the vicinity of the CBM and VBM, within ~0.1 eV to ~0.2 eV. Further away from this energy range, the DFT-TB model gives much higher DOS values, which means that PNRs described by a more detailed Hamiltonian model will provide a higher amount of inversion charge that can be induced by appropriate adjustment of the quasi-Fermi level in the channel by the gate electrode, for both *n*- and *p*-type devices. For the narrowest PNR, the two DOS characteristics diverge considerably even near the CBM and VBM, which is caused by the large difference in m* between the two models. Therefore, we can expect the largest discrepancy in PNR FET performance between the two TB models for 0.49-nm-wide PNR FETs.

**Figure 4:**
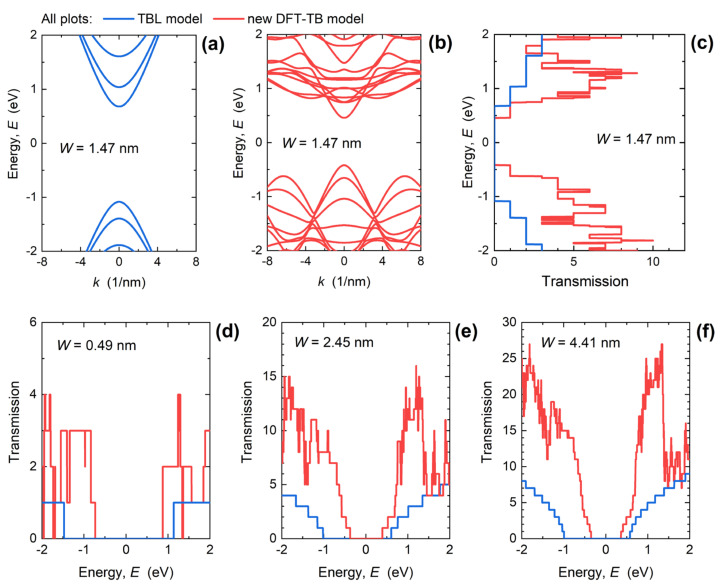
Dispersion obtained by (**a**) TBL and (**b**) DFT-TB model, and (**c**) transmission for the 1.47 nm-wide PNR. Comparison of transmission functions obtained with the two TB Hamiltonian models for *W* of (**d**) 0.49 nm, (**e**) 2.45 nm, and (**f**) 4.41 nm.

Connection between the bandstructure and transmission through the nanoribbon is illustrated in Figure 4a–c for the 1.47 nm-wide PNR. Namely, transmission function counts the number of conducting modes with a positive velocity at a certain energy and, hence, the DFT-TB model gives a generally higher transmission due to richer bandstructure than the TBL model. For *W* = 1.47 nm in Figure 4c, and other PNR widths from 0.49 nm to 4.41 nm reported in Figure 4d–f, we again observe that the TBL model reproduces the transmission well only near the CBM and VBM, while at higher energies *E* > CBM + 0.2 eV, the DFT-TB model provides a more complex characteristic and higher transmission values. Due to the higher number of conducting modes, the improved bandstructure description by the DFT-TB model results in up to ~4× higher transmission in comparison to the TBL model.

As shown in Figure 4, downscaling of *W* leads to an increased transmission gap and a reduced transmission maximum. In the examined energy range, from −2 eV to 2 eV, the transmission maxima decrease from 27, over 16 and 10, to 4 when PNR width decreases from 4.41 nm, over 2.45 nm and 1.47 nm, down to 0.49 nm. At the same time, the transport gap extracted as the energy range where *T*(*E*) < 0.01 increases from 0.71 eV for *W* = 4.41 nm up to 1.61 eV for the 0.49 nm-wide PNR. This increase makes narrower PNRs more immune to tunneling effects, which improves the performance of narrow PNR FETs in the OFF-state. It is expected that the reduced transmission in narrower PNRs will lead to poorer ON-state performance when *W* decreases, but device simulations are needed for a proper assessment due to several competing factors that play a role in device operation. For example, in the case of *W* = 0.49 nm, electrons are considerably heavier than in wider PNRs (Figure 2a), which results in high DOS (Figure 3a), and a high inversion charge density can be expected in 0.49 nm-wide PNR FETs. In addition, the transmission is boosted near the CBM in comparison to the TBL results (Figure 4d), which suggest a high drain current. However, the high effective mass (m*~1.9*m*_0_) points toward a lower carrier velocity and overall poorer current-driving capabilities of 0.49 nm-wide PNR FETs.

### 3.2. Performance of Ultra-Scaled PNR FETs

In this work, we investigate the performance of 15 nm-long PNR FETs, for which we assume SiO_2_ as gate dielectric with a thickness of 1 nm and S/D doping of *m* = 0.001, where *m* is the molar fraction of the areal density of P atoms in PNRs, resulting in a doping density of ~4 × 10^12^ cm^−2^. A common threshold voltage (*V_TH_*) of 0.24 V, as projected in the International Roadmap for Devices and Systems (IRDS) at the “3 nm” CMOS node [39], is set for all devices by automatically adjusting the gate work function. Setting the same *V_TH_*, and consequently the same OFF-state current (*I_OFF_*), allows a meaningful and fair comparison of PNR FETs with different nanoribbon widths. The resulting *I_OFF_* is ~1 nA/µm, defined for *V_DS_* = 0.7 V and *V_GS_* = 0 V. The supply voltage is 0.7 V, so the ON-state current (*I_ON_*) is extracted from *I*-*V* characteristics for *V_DS_* = *V_GS_* = 0.7 V. Average charge velocity (*v_avg_*) at ToB is calculated from the drain current and the obtained ToB charge density. The ON-state ToB charge density (*Q_s_*_,*ON*_) and ON-state velocity (*v_ON_*) are also extracted at the same bias point as *I_ON_*, i.e., with gate and drain biased at the supply voltage.

**Figure 5:**
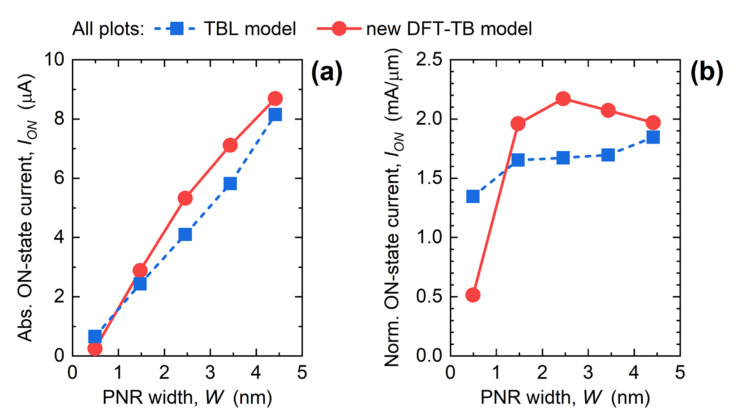
Impact of PNR width downscaling on (**a**) absolute *I_ON_* and (**b**) width-normalized *I_ON_* in PNR FETs. The plots compare the results obtained by DFT-TB and TBL models.

Figure 5 reports the dependence of *I_ON_* on PNR width for both the absolute magnitude of the current (Figure 5a) and for the width-normalized current (Figure 5b). The absolute *I_ON_* monotonically decreases with the downscaling of PNR width, from 8.7 µA in the 4.41 nm-wide PNR FET down to 0.25 µA for *W* = 0.49 nm. In comparison to the TBL model, DFT-TB Hamiltonians with a more accurate PNR bandstructure provide higher driving currents, except for the narrowest device. These *I_ON_* values in single PNRs are too low to be practically relevant, so several PNRs must be connected in parallel to provide sufficiently high *I_ON_*. The plausibility of utilizing PNRs in extremely scaled FETs is quantified by the width-normalized *I_ON_* reported in Figure 5b, which allows an assessment of PNR FET performance against IRDS requirements. As shown previously [22], *I_ON_* obtained by the TBL model exhibits a generally decreasing trend with a weak modulation by W downscaling. Moreover, none of the examined PNR FETs using the TBL model fulfills the IRDS requirement for *I_ON_* at the “3 nm” node, i.e., *I_ON_* > 1.9 mA/µm [39]. In contrast, device simulations using DFT-TB Hamiltonians provide significant qualitative and quantitative changes. First, the *I_ON_* vs. *W* characteristic is non-monotonic and the width-normalized *I_ON_* exhibits a local maximum of 2.17 mA/µm for *W* = 2.45 nm, which means that 2.45 nm-wide PNR FETs are the most area-efficient devices in terms of current drivability. Regarding IRDS requirements, DFT-TB model reveals that PNR FETs with *W* > 1.4 nm can surpass the *I_ON_* target set by the IRDS. The DFT-TB model reveals the severity of bandstructure effects in the narrowest devices because *I_ON_* for *W* = 0.49 nm is only 0.51 mA/µm, which is ~2.7× lower than obtained by the TBL model. Going towards the widest examined nanoribbons, both Hamiltonian models converge to the same *I_ON_*, which is expected as both approaches describe large-area phosphorene equally well.

**Figure 6:**
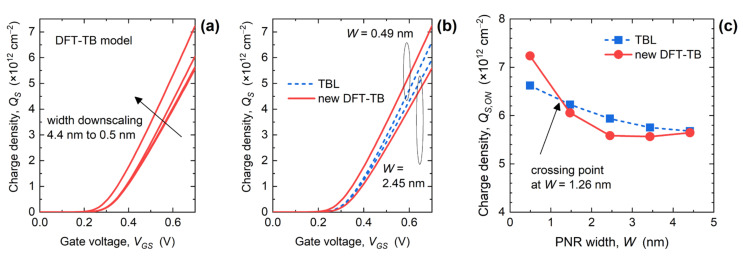
(**a**) Charge density vs. gate bias characteristics in PNR FETs. Comparison of (**b**) *Q_s_*-*V_GS_* curves for 0.49 nm and 2.45 nm-wide devices, and (**c**) *Q_s_*-*W* characteristics in the ON-state obtained by the two TB models.

**Figure 7:**
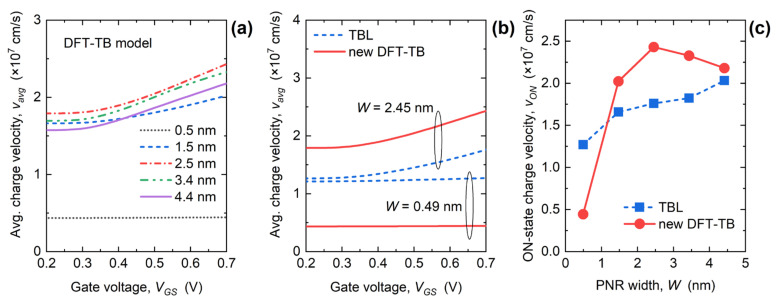
(**a**) Average charge velocity vs. *V_GS_* in PNR FETs for the DFT-TB model. Comparison of (**b**) *v_avg_*-*V_GS_* curves for 0.49 nm and 2.45 nm-wide PNR FETs, and (**c**) ON-state *v_avg_*-*W* characteristics obtained by the DFT-TB and TBL models.

The size-scaling bandstructure effects on the device performance can be more easily understood by exploring the size- and bias-dependent properties of charge density (Figure 6) and average charge velocity (Figure 7) in the channel of PNR FETs. As shown in Figure 6a, *Q_s_* increases with increasing *V_GS_* up to ~7 × 10^12^ cm^−2^, and generally increases with the downscaling of PNR width, although *Q_s_*-*V_GS_* curves are closely spaced for *W* > 1.5 nm. This *Q_s_* behavior is attributed to the increasing DOS near the CBM when *W* decreases (see Figure 3). Figure 6b compares the bias-dependence of *Q_s_* for the two TB models for 0.49 nm and 2.45 nm-wide phosphorene nanodevices. Clearly, the TBL model overestimates the channel charge density for *W* = 2.45 nm and underestimates it for *W* = 0.49 nm. The impact of width downscaling on *Q_s_* in the ON-state (*Q_s_*_,*ON*_) is reported in Figure 6c, and we observe that both models result in monotonic increase of *Q_s_*_,*ON*_ when *W* decreases. The DFT-TB model provides a somewhat stronger modification of charge density with *W*, and the *Q_s_*_,*ON*_ from DFT-TB simulations surpasses the TBL model results only for *W* < 1.26 nm. This result for *W* = 0.49 nm is expected because, from Figure 2a, it is clear that DFT-TB bandstructure exhibits heavier electrons in the lowest subbands and a generally higher number of subbands near the CBM when compared to the simpler TBL model. On the other hand, wider PNRs defined by DFT-TB Hamiltonians have slightly lighter carriers, which results in *Q_s_*_,*ON*_ being somewhat lower in the 1.5 nm to 3.5 nm width range. Since the *Q_s_* behavior is in disagreement with *I_ON_* trends, the reasons for improvement introduced by the DFT-TB model reported in Figure 5 must come from the gate capacitance (*C_G_*) or the average charge velocity.

Regarding *C_G_*, it can be assessed as a series of *C_ox_* and quantum capacitance of the channel (*C_q_*) defined as *C_q_* = *d*(*qQ_s_*)/*dV_GS_*. Since gate oxide (SiO_2_) thickness is 1 nm for all devices, *C_ox_* = 34.5 fF/µm^2^ and is independent of PNR width. On the other hand, *C_q_* increases from ~102 fF/µm^2^ for *W* = 4.41 nm up to ~248 fF/µm^2^ (TBL) and ~298 fF/µm^2^ (DFT-TB) for the 0.49 nm-wide device. Therefore, the dependence of *C_q_* is qualitatively the same as *Q_s_* in Figure 6c. In addition, *C_q_* is much larger than *C_ox_*, so the total *C_G_* only slightly increases with the downscaling of PNR width, and the absolute values of *C_G_* are 26–31 fF/µm^2^. If the oxide thickness were to decrease, *C_ox_* would rise, which in turn would enhance the relative impact of *C_q_* in the total *C_G_* of PNR FETs. Consequently, bandstructure effects reported using the DFT-TB model would be more pronounced, but a more detailed investigation is beyond the scope of the current paper.

Figure 7a plots *V_GS_*-dependence of *v_avg_* for *V_DS_* = 0.7 V obtained using the DFT-TB model for various PNR widths. Generally, *v_avg_* increases with increasing *V_GS_*, while for *W* = 0.49 nm the velocity is independent of gate bias. The minimum *v_avg_* of 0.44 × 10^7^ cm/s is reported for the 0.49 nm-wide PNR FET, while the highest velocity is reached for *W* = 2.45 nm irrespective of *V_GS_*. For *W* = 2.45 nm, *v_avg_* equals 1.79 × 10^7^ cm/s at threshold and grows to 2.43 × 10^7^ cm/s in the ON-state. The changes introduced by the improved Hamiltonian model are illustrated in Figure 7b that compares *v_avg_* in 0.49 nm and 2.45 nm-wide nanoribbons calculated using the two TB models. While both models give identical qualitative *v_avg_* behavior with respect to *V_GS_*, the TBL model underestimates the velocity for *W* = 2.45 nm and overestimates it for the 0.49 nm-wide PNR FET. In turn, these characteristics lead to weak *W*-dependence of *I_ON_* for the TBL model reported in Figure 5b. Finally, the influence of PNR width downscaling on *v_ON_* is illustrated in Figure 7c, and the curve exhibits a monotonic *v_ON_* decrease in case of the TBL model. In contrast, a non-monotonic *v_ON_* behavior is observed in case of the DFT-TB model with a maximum *v_ON_* of 2.43 × 10^7^ cm/s recorded in the 2.45 nm-wide PNR FET. This local *v_ON_* maximum for *W* = 2.45 nm is a consequence of two competing mechanisms. As shown in Figure 2 and discussed in the related text, the first-subband effective mass monotonically increases in narrower PNRs, so a monotonic velocity decrease should occur with *W* downscaling. On the other hand, the average charge velocity considers all populated subbands, including higher subbands that exhibit larger effective masses. Therefore, the characteristics of the first subband are important, but not the only one responsible for the overall *v_ON_* behavior. When the PNR width decreases, the separation between subbands increases, which in turn decreases their population and their relative impact on *v_ON_*. Hence, 2.45 nm presents an optimum width where these two mechanisms jointly result in maximum average charge velocity in the ON-state. Finally, by comparing the results in Figure 5b and Figure 7c, we conclude that the *I_ON_* characteristics reported in Figure 5b are dominantly caused by the bias- and width-dependence of carrier velocity in the channel.

## 4. Conclusions

Our results are obtained for ballistic transport across the channel, but in reality, carriers would likely experience scattering by intrinsic or extrinsic scattering centers, such as acoustic and optical phonons, Coulomb centers, defects, etc. [40,41]. Moreover, we neglect contact resistance in this study although it is a severe performance limiter in all 2D material-based FETs [42,43], including monolayer BP [4,44,45] and nanoribbon-based FET structures [22,33,46,47]. Nevertheless, our work illustrated the importance of using a proper Hamiltonian in the simulation of PNR nanodevices, quantified the magnitude of size-scaling and bandstructure effects, and demonstrated a considerable improvement of PNR FET figures-of-merit in comparison to the simpler TBL model. We found that for *W* = 2.45 nm (best device), the PNR FET is able to tolerate 13% current loss due to scattering and still meet the IRDS *I_ON_* target at the “3 nm” technology node. The ballisticity level of 87% seems attainable because in a large-area phosphorene FET with a 15 nm-long channel, the ballisticity of ~90% was reported after including phonon scattering [48]. We note that the impact of electron-phonon scattering on the transport properties and performance of phosphorene nanodevices is under investigation, and the ballisticity level reported in [48] might be lower [18].

Monolayer BP or phosphorene is an attractive candidate for future electronic devices, while nanostructured BP in the form of PNRs offers an additional avenue for adjusting the electronic, transport, and device properties by quantum confinement effects. The nature of nanoscale BP devices demands advanced theoretical approaches based on quantum transport and appropriate atomically-resolved device Hamiltonians. In this work, we explored ultra-narrow PNRs and PNR FETs with the widths under ~5 nm using NEGF simulations based on a recently developed DFT-TB Hamiltonian model that accurately reproduces multi-valley dispersion and valence-conduction band asymmetry observed in PNRs by ab initio calculations. We explored the dispersion, DOS, and transmission through PNRs of various widths and reported a strong impact of width-scaling on the number and shape of conducting subbands, bandgap, and carrier effective mass. Focusing on 15 nm-long ballistic PNR FETs, we found that the ON-state inversion charge density increases up to ~7 × 10^12^ cm^−2^, and that the average charge velocity decreases considerably (from ~2.2 × 10^7^ cm/s to ~0.4 × 10^7^ cm/s) with the downscaling of PNR width. Velocity decrease was found to be the dominant factor in current-driving properties, so the ON-state current in PNR FETs also declines in narrower nanoribbons, from ~2 mA/µm (*W* = 4.41 nm) to ~0.5 mA/µm (*W* = 0.49 nm). Nevertheless, using an improved bandstructure description with the DFT-TB model revealed that ballistic PNR FETs with *W* > 1.4 nm can meet the IRDS requirement for *I_ON_* at the “3 nm” CMOS technology node. Moreover, an optimum PNR FET with *W* = 2.45 nm was found, which exhibits *I_ON_* ~ 2.2 mA/µm and which can operate at 87% of the ballistic limit and still meet the IRDS target for the ON-state current.

## Data Availability

The data presented in this study are contained within the article and are available on request from the corresponding author.

## Supplementary Materials

The following supporting information can be downloaded at https://www.mdpi.com/article/10.3390/ma15010243/s1, File S1: compressed folder containing the tight-binding model data (matrices) for the construction of nanoribbon Hamiltonians.
